# *IQSEC2*-related encephalopathy in males and females: a comparative study including 37 novel patients

**DOI:** 10.1038/s41436-018-0268-1

**Published:** 2018-09-12

**Authors:** Cyril Mignot, Aoife C. McMahon, Claire Bar, Philippe M Campeau, Claire Davidson, Julien Buratti, Caroline Nava, Marie-Line Jacquemont, Marilyn Tallot, Mathieu Milh, Patrick Edery, Pauline Marzin, Giulia Barcia, Christine Barnerias, Claude Besmond, Thierry Bienvenu, Ange-Line Bruel, Ledia Brunga, Berten Ceulemans, Christine Coubes, Ana G. Cristancho, Fiona Cunningham, Marie-Bertille Dehouck, Elizabeth J. Donner, Bénédicte Duban-Bedu, Christèle Dubourg, Elena Gardella, Julie Gauthier, David Geneviève, Stéphanie Gobin-Limballe, Ethan M. Goldberg, Eveline Hagebeuk, Fadi F. Hamdan, Miroslava Hančárová, Laurence Hubert, Christine Ioos, Shoji Ichikawa, Sandra Janssens, Hubert Journel, Anna Kaminska, Boris Keren, Marije Koopmans, Caroline Lacoste, Petra Laššuthová, Damien Lederer, Daphné Lehalle, Dragan Marjanovic, Julia Métreau, Jacques L. Michaud, Kathryn Miller, Berge A. Minassian, Joannella Morales, Marie-Laure Moutard, Arnold Munnich, Xilma R. Ortiz-Gonzalez, Jean-Marc Pinard, Darina Prchalová, Audrey Putoux, Chloé Quelin, Alyssa R. Rosen, Joelle Roume, Elsa Rossignol, Marleen E. H. Simon, Thomas Smol, Natasha Shur, Ivan Shelihan, Katalin Štěrbová, Emílie Vyhnálková, Catheline Vilain, Julie Soblet, Guillaume Smits, Samuel P. Yang, Jasper J. van der Smagt, Peter M. van Hasselt, Marjan van Kempen, Sarah Weckhuysen, Ingo Helbig, Laurent Villard, Delphine Héron, Bobby Koeleman, Rikke S. Møller, Gaetan Lesca, Katherine L. Helbig, Rima Nabbout, Nienke E. Verbeek, Christel Depienne

**Affiliations:** 1 INSERM, U 1127, CNRS UMR 7225, Sorbonne Universites, UPMC Univ Paris 06 UMR S 1127, Institut du Cerveau et de la Moelle epiniere, ICM, Paris, France; 2 APHP, Hôpital Pitie-Salpetriere, Departement de Genetique et de Cytogenetique; Centre de Reference Deficience Intellectuelle de Causes Rares; GRC UPMC «Deficience Intellectuelle et Autisme», Paris, France; 3 European Molecular Biology Laboratory, European Bioinformatics Institute, Wellcome Genome Campus, Hinxton, Cambridge UK; 4 APHP, Reference Centre for Rare Epilepsies, Necker-Enfants Malades Hospital, Imagine Institute, Paris Descartes University, Paris, France; 5 INSERM U1163, Imagine Institute, Paris, France; 6 Paris Descartes University, Paris, France; 7 Division of Medical Genetics, Department of Pediatrics, CHU Sainte-Justine and University of Montreal, Montreal, QC Canada; 8 CHU La Reunion–Groupe Hospitalier Sud Reunion, La Reunion, France; 9 APHM, Hôpital d’Enfants de La Timone, Service de Neurologie Pediatrique, centre de reference deficiences intellectuelles de cause rare, Marseille, France; 10 Aix Marseille University, INSERM, MMG, UMR-S 1251, Faculte de medecine, Marseille, France; 11 Service de Genetique, Centre de Reference Anomalies du Developpement, Hospices Civils de Lyon, Bron, France; 12 INSERM U1028, CNRS UMR5292, Centre de Recherche en Neurosciences de Lyon, GENDEV Team, Universite Claude Bernard Lyon 1, Bron, France; 13 Claude Bernard Lyon I University, Lyon, France; 14 APHP, Service de genetique medicale, Necker-Enfants Malades Hospital, Imagine Institute, Paris Descartes University, Paris, France; 15 APHP, Unite fonctionnelle de Neurologie, Necker-Enfants Malades Hospital, Imagine Institute, Paris Descartes University, Paris, France; 16 APHP, Laboratoire de Genetique et Biologie Moleculaires, Hôpital Cochin, HUPC, Paris, France; 17 Universite Paris Descartes Paris, Institut de Psychiatrie et de Neurosciences de Paris, Inserm U894, Paris, France; 18 FHU-TRANSLAD, Universite de Bourgogne/CHU Dijon, Dijon, France; 19 INSERM UMR 1231 GAD team, Genetics of Developmental disorders, Universite de Bourgogne-Franche Comte, Dijon, France; 20 Division of Neurology, Department of Paediatrics, The Hospital for Sick Children, University of Toronto, Toronto, ON Canada; 21 Department of Pediatric Neurology, University Hospital and University of Antwerp, Antwerp, Belgium; 22 Departement de Genetique Medicale, Maladies rares et Medecine Personnalisee, CHU de Montpellier, Montpellier, France; 23 Division of Neurology, Children’s Hospital of Philadelphia, Philadelphia, PA USA; 24 Centre de Genetique Chromosomique, Hôpital St-Vincent-de-Paul, GHICL, Lille, France; 25 CHU Rennes, Service de Genetique Moleculaire et Genomique, Rennes, France; 26 Danish Epilepsy Centre Filadelfia, Dianalund, Denmark; 27 Institute for Regional Health Services, University of Southern Denmark, Odense, Denmark; 28 INSERM U1183, Montpellier, France; 29 Stichting Epilepsie Instellingen Nederland, SEIN, Zwolle, The Netherlands; 30 Department of Biology and Medical Genetics, Charles University 2nd Faculty of Medicine and University Hospital Motol, Prague, Czech Republic; 31 APHP, University Hospital of Paris ïle-de-France ouest, Raymond Poincare Hospital, Garches, France; 32 Department of Clinical Diagnostics, Ambry Genetics, Aliso Viejo, CA USA; 33 Centre for Medical Genetics Ghent, Ghent University Hospital, C. Heymanslaan 10, Ghent, Belgium; 34 Service de Genetique Medicale, Hôpital Chubert, Vannes, France; 35 APHP, Department of Clinical Neurophysiology, Necker-Enfants Malades Hospital, Paris, France; 36 Department of Genetics, University Medical Center Utrecht, Utrecht, The Netherlands; 37 Departement de Genetique Medicale, APHM, Hopital d’Enfants de La Timone, Marseille, France; 38 Child Neurology Department, 2nd Faculty of Medicine, Charles University and Motol Hospital, Prague, Czech Republic; 39 Centre de Genetique Humaine, Institut de Pathologie et de Genetique, Gosselies, Belgium; 40 Unite fonctionnelle de genetique clinique, Centre Hospitalier Intercommunal de Creteil, Creteil, France; 41 APHP, Service de neurologie pediatrique, Hôpital Universitaire Bicetre, Le Kremlin-Bicetre, France; 42 Department of Pediatrics, Albany Medical Center, Albany, NY USA; 43 APHP, Hôpital Trousseau, service de neuropediatrie, Paris, France; 44 Sorbonne Universite, GRC n°19, pathologies Congenitales du Cervelet-LeucoDystrophies, APHP, Hôpital Armand Trousseau, Paris, France; 45 Division of Neuropediatrics, CHU Raymond Poincare (APHP), Garches, France; 46 Service de Genetique Medicale, CLAD Ouest CHU Hôpital Sud, Rennes, France; 47 Unite de Genetique Medicale, Centre de Reference des Maladies rares du Developpement (AnD DI Rares), CHI Poissy–St Germain en Laye, Poissy, France; 48 Departments of Pediatrics and Neurosciences, CHU Sainte-Justine and University of Montreal, Montreal, Canada; 49 Institut de Genetique Medicale, CHRU Lille, Universite de Lille, Lille, France; 50 Department of Genetics, Hôpital Universitaire des Enfants Reine Fabiola, ULB Center of Human Genetics, Universite Libre de Bruxelles, Brussels, Belgium; 51 Department of Genetics, Hôpital Erasme, ULB Center of Human Genetics, Universite Libre de Bruxelles, Brussels, Belgium; 52 Interuniversity Institute of Bioinformatics in Brussels, Universite Libre de Bruxelles, Brussels, Belgium; 53 Clinical Genomics & Predictive Medicine, Providence Medical Group, Dayton, WA USA; 54 Department of Metabolic Diseases, Wilhelmina Children′s Hospital, University Medical Center, Utrecht, The Netherlands; 55 Neurogenetics Group, Center of Molecular Neurology, VIB, Antwerp, Belgium; 56 Neurology Department, University Hospital Antwerp, Antwerp, Belgium; 57 IGBMC, CNRS UMR 7104/INSERM U964/Universite de Strasbourg, Illkirch, France; 58 Institute of Human Genetics, University Hospital Essen, University of Duisburg-Essen, Essen, Germany

**Keywords:** *IQSEC2*, X-linked inheritance, epilepsy, intellectual disability, isoforms

## Abstract

**Purpose:**

Variants in *IQSEC2*, escaping X inactivation, cause X-linked intellectual disability with frequent epilepsy in males and females. We aimed to investigate sex-specific differences.

**Methods:**

We collected the data of 37 unpublished patients (18 males and 19 females) with *IQSEC2* pathogenic variants and 5 individuals with variants of unknown significance and reviewed published variants. We compared variant types and phenotypes in males and females and performed an analysis of *IQSEC2* isoforms.

**Results:**

*IQSEC2* pathogenic variants mainly led to premature truncation and were scattered throughout the longest brain-specific isoform, encoding the synaptic IQSEC2/BRAG1 protein. Variants occurred de novo in females but were either de novo (2/3) or inherited (1/3) in males, with missense variants being predominantly inherited. Developmental delay and intellectual disability were overall more severe in males than in females. Likewise, seizures were more frequently observed and intractable, and started earlier in males than in females. No correlation was observed between the age at seizure onset and severity of intellectual disability or resistance to antiepileptic treatments.

**Conclusion:**

This study provides a comprehensive overview of *IQSEC2-*related encephalopathy in males and females, and suggests that an accurate dosage of IQSEC2 at the synapse is crucial during normal brain development.

## Introduction

Pathogenic variants on chromosome X represent an important cause of neurodevelopmental disorders, where expression usually depends on the sex of the individual.^1^ X-linked conditions are often restricted to males, or more severe in males than in females, because males have a single copy of the X chromosome while females have two copies.^2^ *EFNB1-*related craniofrontonasal syndrome and *PCDH19-*related female-limited epilepsy, two X-linked disorders where heterozygous females are affected and hemizygous males are spared, constitute the only exceptions to this rule known so far.^3,4^ Yet, increasing evidence shows that variants on chromosome X also account for a significant proportion of affected females and that some X-linked disorders affect males and females almost equally. Compensation of gene dosage in females is achieved for most genes on the X chromosome by epigenetic silencing of one X chromosome. This process, known as X chromosome inactivation (XCI), usually occurs randomly from one cell to another at an early stage of embryonic development. XCI is stably inherited over subsequent cell divisions, which ultimately results in somatic mosaicism at the organism level where statistically half of the cells inactivate the paternal-derived X chromosome whereas the other half inactivate the maternal copy.^2,5^ In females carrying a pathogenic X-linked variant, skewing of XCI favoring the expression of the normal allele protects from the disorder while skewing favoring the expression of the mutated allele has the opposite effect.^6^ Consequently, females with X-linked neurodevelopmental disorders usually have more variable phenotypes assumed to be related to XCI patterns in the brain. Interestingly, 15–20% of genes escape XCI and are expressed from both X alleles in females.^7,8^ Escapee genes differ from one mammalian species to the other and both their function and link with human disorders are still largely unknown.^9,10^

The *IQSEC2* gene on chromosome Xp11.22 encodes a guanine nucleotide exchange factor (GEF) able to activate small GTPases of the ARF family, including ARF6^11,12^. Enriched at the postsynaptic density of excitatory synapses where it interacts with proteins of the PSD-95 complexes including PSD-95 itself,^12–15^ the IQSEC2 (IQ motif and SEC7 domain-containing protein 2) protein acts on shaping dendritic spine morphology^16^ and controls excitatory synaptic transmission by regulating glutamate (*N*-methyl-D-aspartate [NMDA] and α-amino-3-hydroxy-5-methyl-4-isoxazolepropionic acid [AMPA]) receptor-mediated responses.^17–19^ *IQSEC2* missense variants were first identified in four large families, in which almost exclusively males were affected by nonsyndromic intellectual disability (ID).^11^ Although a de novo translocation interrupting *IQSEC2* had been described earlier in a female patient,^20^ the recognition that *IQSEC2* encephalopathy also affects females came from the further identification of de novo *IQSEC2* variants in females with epileptic encephalopathy or ID.^1,21–29^ Recently, two families with affected males and females,^18,30^ and one family with affected females only, probably as the result of paternal gonadal mosaicism,^31^ were also described.

Remarkably, *IQSEC2* is one of the few genes that escape XCI in humans.^7^ This property is specific to the human or primate lineage because *Iqsec2*, although located in the homologous region of chromosome X, is conversely subject to XCI in mice.^32^ Moreover, *IQSEC2* leads to the expression of at least three different isoforms. The nature of the isoforms that are relevant for the human disorder and the mechanisms by which the variants lead to a disorder in males and females remain mysterious.

In this study, we compared the variant types and clinical phenotypes of males and females with *IQSEC2* pathogenic variants to gain insights into the mechanisms controlling phenotypic variability. To this end, we collected unreported patients with likely pathogenic variants from different clinical centers and reviewed patients published in the literature.

## Materials and methods

### Human subjects

We collected clinical and molecular data of 47 patients with IQSEC2 variants from different clinical centers in France, Belgium, the Netherlands, Denmark, Czech Republic, Canada, and the United States through clinical and genetics networks (Defiscience, AnDDIrare, EuroEPINOMICS consortium). Variants of five patients (patients 10, 12, 22, 30, and 37) were previously reported without clinical data.^28,33^ Other patients were novel. Details on the methods used to detect the *IQSEC2* variants are indicated for each patient in Table S1. The American College of Medical Genetics and Genomics (ACMG) criteria and the Intervar interface (http://wintervar.wglab.org/) were used to filter and classify *IQSEC2* variants.^34,35^ Each referring physician filled out a table with detailed developmental, neurological, behavioral, and epileptic medical history, including electroencephalogram (EEG) and imaging data where available. Patients were evaluated according to developmental scales routinely used in enrolled centers (Brunet–Lezine, HAWIK-IV, SON-R2). Informed written consent was locally obtained for all participants. The study was approved by INSERM (RBM C12-06).

### Review of published *IQSEC2* variants

**Fig. 1:**
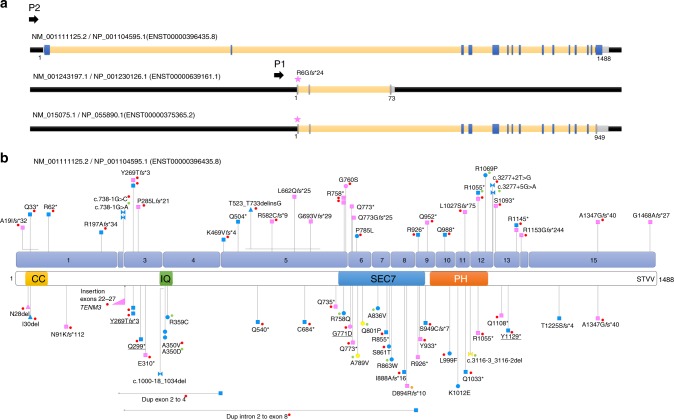
**Schematic representation of** ***IQSEC2*** **isoforms and location of pathogenic** ***IQSEC2*** **variants on the longest isoform.** (**a**) Schematic representation of the three *IQSEC2* isoforms resulting from alternative promoter usage (P1 vs. P2) and splicing. Blue/gray boxes respectively indicate exons present/absent in the longest isoform. The pink star indicates the location of the *IQSEC2* variant (unknown significance) identified in patient 47. (**b**) Location of *IQSEC2* pathogenic variants reported in this study (above) or in the literature (below) on the schematic representation of the longest NM_001111125.2 isoform (numbered blue boxes correspond to exons) and corresponding protein domains: N-terminal coiled coil (CC) domain, IQ calmodulin-binding motif (IQ), SEC7 and Pleckstrin homology (PH) domains, and PDZ-binding motif (STVV). Square: predicted truncating variants; circle: missense variants; triangle: in-frame deletion; bow tie: splice variants; pink: variants present in affected females; blue: variants present in affected males; yellow: variants present in affected males and females. Red dot: de novo occurrence; green dot: maternal inheritance; orange dot: suspected gonadal mosaicism. No dot indicates inheritance is unknown. Horizontal lines indicate the extent of large deletions on the corresponding coding sequence. Variants underlined: previously published without clinical data and included in this study. Variants of unknown significance (VUS) or variants from the literature for which sex was unknown are not indicated on this schematic

The terms “IQSEC2” and “variants” or “mutations” were used to search for articles reporting *IQSEC2* patients in PubMed. In addition, variants present in the Human Gene Mutation Database (HGMD) professional database (Biobase, Qiagen) were reviewed. We contacted the authors of the article when information on sex of the patient and inheritance/status of the variant was unavailable or to rule out the possibility that patients with identical variants are duplicates. The accuracy of variant description was checked using Alamut 2.10 (Interactive Biosoftware). Deletions encompassing *IQSEC2* and additional genes were excluded from the review. *IQSEC2* variants are indicated on the longest isoform (complementary DNA: NM_001111125.2/ENST00000396435.8; protein: NP_001104595.1) unless specified differently, according to Human Genome Variation Society (HGVS) guidelines (www.hgvs.org/mutnomen). Pathogenic variants and corresponding patient data (sex, status, variant inheritance) were listed (Table S2) and visualized on the schematic representation of *IQSEC2* (Fig. 1b).

### *IQSEC2* isoforms

A manual review of the existing GENCODE (ENST) and RefSeq (NM) annotation of the IQSEC2 locus (GRCh38) was performed collaboratively by an LRG project curator and a GENCODE annotator and found to be satisfactory (pending LRG_1194). The shortest isoform (ENST00000639161.1/NM_001243197.1) was not yet annotated in the GRCh37/hg19 genome annotation used by most publicly available transcript quantification projects (e.g., GTex), so isoform expression was determined using alternative methods. Promoter usage was determined using Fantom5 Cap Analysis Gene Expression (CAGE) data from the Riken consortium, accessed using the SSTAR portal (TSS expression).^36^ Isoform specific expression was determined by quantifying intron-spanning reads of isoform specific exon–exon boundaries in publicly available RNA-seq data sets (accessed through ENA). Exon–exon boundaries quantified were (GRCh38 coordinates): NM_001111125.2/ENST00000396435.8, introns 1 (chrX:53291925:53320416) and 2 (chrX:53256062:53291894); NM_015075.1/ENST00000375365.2, intron 2 (chrX:53256062:53279563); NM_001243197.1/ENST00000639161.1, intron 2 (chrX_53267065:53279563). Additional information is provided in Table S3.

### Genotype–phenotype correlations

Means and frequencies were compared using the Fisher’s exact test or Wilcoxon–Mann–Whitney test where appropriate. Comparative violin plots were drawn using Seaborn, a Python visualization library based on Matplotlib (https://seaborn.pydata.org/index.html).

## Results

We collected the data from 37 unpublished patients (18 males and 19 females) with *IQSEC2* pathogenic variants, 5 individuals (4 males and 1 female) with variants previously reported without clinical data, and 5 novel patients (3 males and 2 females) with variants of unclear significance (VUS) from 45 unrelated families (Table 1): one splice-site variant inherited from an asymptomatic mother was present in two brothers (patients 5 and 6) and a missense VUS was present in a male and his maternal uncle (patients 45 and 46; Table S1, Fig. 1). We also reviewed information from 100 carrier individuals from 52 published families (Table S2). The sex ratio for affected individuals from the literature was 54 males to 23 females (sex unknown for 9 affected individuals). A total of 15 unaffected heterozygous female carriers from 4 families were also described.^11^

### *IQSEC2* isoforms

The longest *IQSEC2* isoform (NM_001111125.2/NP_001104595.1) comprises 15 exons and encodes a 1488 amino acid (aa) protein (Fig. 1a, b). The corresponding protein is part of the PSD-95 multiprotein complexes in glutamatergic synapses.^14,15^ It contains an N-terminal coiled coil (CC) domain, an IQ calmodulin-binding motif, SEC7 and Pleckstrin homology (PH) domains, as well as a 4-aa PDZ-binding motif (STVV) located at the C-terminus and required for interaction with synaptic PDZ proteins (PSD-95, SAP102, MAGI1, MAGI2).^13^ This protein has also been described in the literature as BRAG1 (brefeldin A resistant Arf-guanine nucleotide exchange factor 1).

**Fig. 2:**
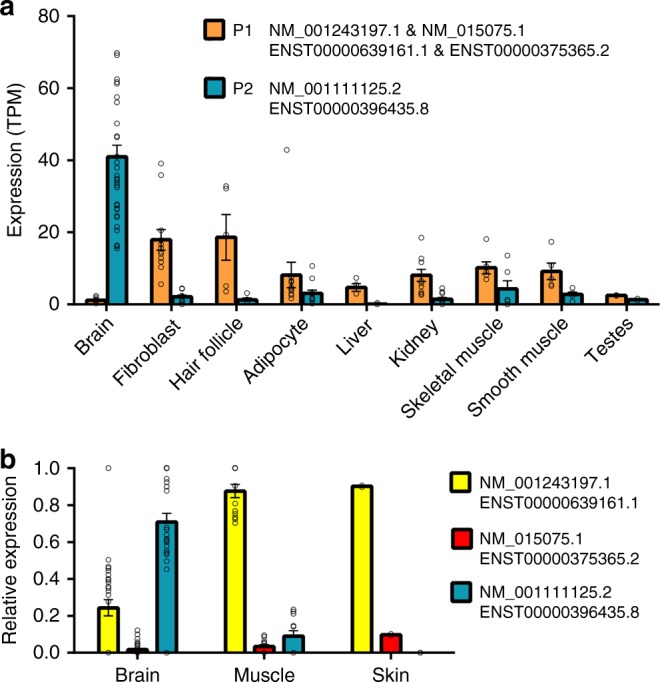
**The PDZ-binding domain IQSEC2 isoform (NM_001111125.2 / NP_001104595.1) is the dominant form expressed specifically in brain.** Expression of IQSEC2 isoforms across multiple tissues was quantified using two different methods. (**a**) Cap Analysis of Gene Expression (CAGE) data shows that the upstream transcription start site (P2), which gives rise only to NM_001111125.2/ENST00000396435.8, is strongly and specifically expressed in the brain while the downstream transcription start site (P1) is more broadly expressed across other tissues. Tissues were chosen based on relatively strong expression of any TSS and sample data, then grouped by tissue. (**b**) Isoform specific intron-spanning RNA-seq reads were quantified to show relative levels of isoform expression, again confirming that NM_001111125.2 is the dominant brain form. Between the two non-PDZ-binding domain forms (NM_001243197.1/ENST00000639161.1 and NM_015075.1/ENST00000375365.2) the shortest form NM_001243197.1 is the isoform predominantly expressed in nonneuronal tissues, and it is also weakly expressed in the brain. Tissues were chosen based on Fig. 2a, but are more limited due to data availability. All data are mean +/− SEM, individual sample data are provided in Table S3

Our analysis of large-scale expression data sets (GTex, Fantom) confirms that the use of the upstream promoter (P2), which gives rise only to the longest isoform (NM_001111125.2/NP_001104595.1), is the dominant form expressed across multiple brain tissues (Fig. 2a).

Two additional isoforms arising through the use of an alternative downstream promoter (P1) and including alternative 5′ exons exist (Fig. 1a). The shortest isoform (NM_001243197.1/NP_001230126.1) does not share any exons with the longest isoform and encodes an entirely distinct 73 aa protein containing a poly-E motif with homology to cobalamin biosynthesis domain. The coding sequence of this unstudied protein is conserved in mammals, suggesting that it has an important, although possibly completely distinct, function and localization (Fig. S1). The third isoform (NM_015075.1/NP_055890.1) is a combination of 5′ exons from the shortest isoform and 3′ exons from the longest isoform. This results in a 949 residue protein that also lacks the PDZ-binding domain, but does contain IQ, Sec7, and PH domains in common with the longest form (Fig. 1a). Both of these non-PDZ containing isoforms are broadly expressed across a range of tissues but at a relatively low level in the brain (Fig. 2a). Of the two, the previously unstudied, shortest isoform (NM_001243197.1/NP_001230126.1) is much more highly expressed in the brain and other tissues than the intermediate NM_015075.1/NP_055890.1 form (Fig. 2b).

All pathogenic *IQSEC2* variants from our series and the literature were scattered throughout the exons of the longest brain-specific NM_001111125.2/NP_001104595.1 isoform (Fig. 1b), confirming that the disorder is mainly due to the alteration of the synaptic IQSEC2/BRAG1 protein. Interestingly however, a female individual with developmental delay, ID, stereotypies, and pharmacoresistant seizures had a frameshift deletion located in the alternative exon 1 (NM_015075.1:c.12del, NP_055890.1:p.Arg6Glyfs*24, patient 47), therefore altering only the shorter isoforms (NM_015075.1 and NM_001243197.1, Fig. 1a), which was also present in her unaffected mother (Table S1). This variant was considered as a VUS, based on the maternal inheritance and unusual location restricted to the short isoforms.

### *IQSEC2* variant spectrum

Overall, most pathogenic variants identified in our patient series are predicted to lead to premature termination codons (*n* = 35/40; including 15 frameshifts; 16 nonsense; 4 splice-site variants) altering exons of the long isoform (NM_001111125.2/ NP_001104595.1, Fig. 1b). Only four novel missense variants, three in the SEC7 domain of IQSEC2 (p.Gly760Ser and p.Gly771Asp in females 21 and 22, and p.Pro785Leu in male 25) and one in the PH domain (p.Arg1069Pro in male 33) were classified as likely pathogenic based on their de novo occurrence and/or their predicted in silico damage. Additionally, a de novo in-frame deletion, leading to the loss of 211 amino acids and the insertion of a glycine in exon 5, was classified as pathogenic (p.Thr523_Thr733delinsGly in male 24). In addition to the VUS altering the short isoforms, three missense variants, located in exons 9 (p.Val918Phe in male 43), 13 (p.Ala1139Thr in female 44) and 14 (p.Arg1155Trp in a male and his affected uncle, patients 45 and 46) of the long isoform were classified as VUS due to unusual or mild phenotypes and/or insufficient evidence for pathogenicity. The observation of females with frameshift variants leading to a late C-terminal truncation of the protein indicates that the four last amino acids, necessary for the interaction with PDZ proteins, are required for the function of the protein at the synapse.

Excluding previously published variants and VUS, pathogenic variants mainly lead to premature truncation in females (18 truncating, 1 missense) while males had a broader range of variant types (10 truncating, 4 splice, 3 missense, 1 in-frame deletion). This tendency was confirmed taking data from the literature into account where missense variants were more frequent in males (13 truncating, 2 splice, 11 missense, 1 in-frame deletion) than in females (11 truncating, 1 missense, 1 in-frame deletion; combined *p* value = 0.003, Fisher’s exact test).

In our series, all pathogenic variants for which inheritance could be investigated occurred de novo in females (12/12 individuals). In contrast, males had either de novo (*n* = 12/16) or maternally inherited (4/16) pathogenic variants. Including previously reported individuals, 11/12 variants identified in females occurred de novo with a gonadal mosaicism suspected in the remaining family with multiple affected females; in males, 13 variants occurred de novo while 8 were maternally inherited. Overall, missense variants were therefore more frequently inherited (8/14) than de novo (6/14). Conversely, truncating variants were predominantly de novo (42/44 for which inheritance could be determined; *p* = 5.6E-5, Fisher’s exact test). Variants altering splice sites were either de novo (*n* = 2) or inherited (*n* = 3) in males. Altogether, this indicates that pathogenic *IQSEC2* variants are inherited in 30% of male patients (12/40) and de novo in the remaining 70%, and that missense variants are more frequently inherited than truncating variants, possibly because they are better tolerated in female carriers than truncating variants.

XCI status was studied from genomic DNA extracted from blood in six affected female patients and the three asymptomatic female carriers with heterozygous VUS (Table S1). Four of five affected females had random XCI patterns while two showed skewed (100:0 and 80:20) XCI. The mother of the male patient with p.Val918Phe had a 80:20 XCI pattern while two female carriers with p.Arg1155Trp respectively had 38:62 and 7.6:92.4 XCI ratios.

### Neurodevelopment

**Fig. 3:**
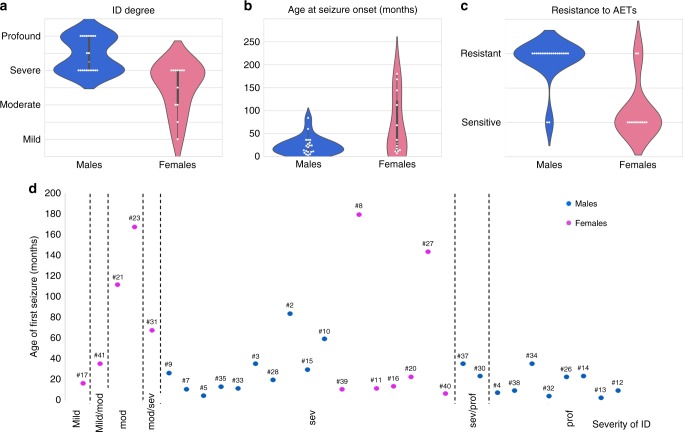
**Comparison of ID severity, seizure onset, and pharmacoresistance in males and females with** ***IQSEC2*** **variants.** (**a**) Violin plots showing the distribution of intellectual disability (ID) degree in males (blue) and females (pink). The distribution of males is clearly shifted to the most severe end of the spectrum while cognitive impairment is on average less severe, but also more variable, in females. (**b**) Violin plots comparing the age at seizure onset in months (*y*-axis) in males (blue) and females (pink), illustrating that epilepsy tends to appear later in females. (**c**) Violin plots comparing the resistance to antiepileptic treatments (AETs) (*y*-axis) in males (blue) and females (pink), showing that epilepsy in males are on average more pharmacoresistant. (**d**) Absence of clear correlation between the degree of comparing the (*y*-axis) and age at seizure onset (*x*-axis). Blue circles: males; pink circles: females; mod: moderate; sev: severe; pro: profound. The numbers above indicate the patient ID number

The study includes 20 females and 22 males with pathogenic *IQSEC2* variants aged from 1.4 to 43 years (Tables S1 and 2). Most patients had normal pregnancy, delivery, and neonatal examination. All patients had developmental delay with ID ranging from mild to profound. Males had severe to profound ID while females had mild to severe ID (mild to moderate, *n* = 6/17; Fig. 3a). Developmental milestones were delayed in the first years of life in most patients, and the delay was significantly more severe in males than in females (mean age at sitting/walking unsupported: 10.2/24.5 months in females versus 20.2/30.3 in males; Table 2). Overall, of the 17 patients older than 3 years who were unable to walk unsupported, one was a female while all others were males. Data on language acquisitions, available for 41 patients, also revealed that male patients had a more severe language delay than female patients, with 20/22 males versus 4/19 females being nonverbal (*p* = 6.2E^−6^, Fisher’s exact test).

**Table 1:** Details of the *IQSEC2* variants identified in the 47 patients reported in this study

Patient ID	Sex M/F	Variant position (hg19)	Change (NM_001111125.2)	CADD score (PHRED)	Prediction SIFT/Polyphen-2 (missense)	Protein domain (missense)	Inheritance	Adjusted ACMG variant classification (InterVar)	Novel (n)/ Reference
1	F	g.53350171_53350267delinsAT	c.55_151delinsAT, .(Ala19Ilefs*32)	–			De novo^a^	Pathogenic	n
2	M	g.53350225G>A	c.97C>T, p.(Gln33*)	36			De novo^a^	Pathogenic	n
3	M	g.53350138G>A	c.184C>T, p.(Arg62*)	35			De novo^a^	Pathogenic	n
4	M	g.53349712_53349734del	c.588_610del, p.(Arg197Alafs*34)	–			De novo^a^	Pathogenic	n
5	M	g.53285244C>T	c.738-1G>A, p.?	25.4			Maternal	Pathogenic	n
6	M	g.53285244C>T	c.738-1G>A, p.?	25.4			Maternal	Pathogenic	n
7	M	g.53285244C>G	c.738-1G>C, p.?	25.2			De novo	Pathogenic	n
8	F	g.53285177del	c.804delC, p.(Tyr269Thrfs*3)	–			De novo^a^	Pathogenic	n
9	M	g.53285177del	c.804delC, p.(Tyr269Thrfs*3)	–			De novo	Pathogenic	n
10	M	g.53285177del	c.804delC, p.(Tyr269Thrfs*3)	–			De novo^a^	Pathogenic	^33^
11	F	g.53285127del	c.854del, p.(Pro285Leufs*21)	32			De novo^a^	Pathogenic	n
12	M	g.53285086G>A	c.895C>T, p.(Gln299^*^)	35			De novo^a^	Pathogenic	^33^
13	M	g.53280352_53280353del	c.1405_1406del, p.(Lys469Valfs*4)	–			De novo^a^	Pathogenic	n
14	M	g.53280248G>A	c.1510C>T, p.(Gln504*)	37			Unknown	Pathogenic	n
15	M	g.53279559_53280191delinsGCC	c.1567_2199delinsGGC, p.(Thr523_Thr733delinsGly)	–			De novo^a^	Pathogenic	n
16	F	g.53279995_53280014del	c.1744_1763del, p.Arg582Cysfs*9	–			De novo	Pathogenic	n
17	F	g.53279758_53279776del	c.1983_1999del, p.(Leu662Glnfs*25)	–			Unknown	Pathogenic	n
18	F	g.53279680del	c.2078delG, p.(Gly693Valfs*29)	–			Unknown	Pathogenic	n
19	F	g.53279486G>A	c.2272C>T, p.(Arg758*)	35			De novo	Pathogenic	n
20	F	g.53279486G>A	c.2272C>T, p.(Arg758*)	35			De novo^a^	Pathogenic	n
21	F	g.53279480C>T	c.2278G>A, p.(Gly760Ser)	32	Tolerated/probably damaging	SEC7	De novo^a^	Likely pathogenic	n
22	F	g.53278050C>T	c.2312G>A, p.(Gly771Asp)	29.1	Deleterious/probably damaging	SEC7	De novo^a^	Likely pathogenic	^28^
23	F	g.53278045G>A	c.2317C>T, p.(Gln773*)	41			Unknown	Pathogenic	n
24	F	g.53278030_53278045del	c.2317_2332del, p.(Gln773Glyfs*25)	–			Unknown	Pathogenic	n
25	M	g.53278008G>A	c.2354C>T, p.(Pro785Leu)	29.8	Deleterious/possibly damaging	SEC7	De novo	Likely pathogenic	n
26	M	g.53272627G>A	c.2776C>T, p.(Arg926*)	40			De novo^a^	Pathogenic	n
27	F	g.53272549G>A	c.2854C>T, p.(Gln952*)	41			De novo^a^	Pathogenic	n
28	M	g.53271019G>A	c.2962C>T, p.(Gln988*)	42			De novo^a^	Pathogenic	n
29	F	g.53268413del	c.3079delC, p.(Leu1027Serfs*75)	–			De novo	Pathogenic	n
30	M	g.53267441G>A	c.3163C>T, p.(Arg1055*)	41			Maternal	Pathogenic	^33^
31	F	g.53267441G>A	c.3163C>T, p.(Arg1055*)	41			Unknown	Pathogenic	n
32	M	g.53267441G>A	c.3163C>T, p.(Arg1055*)	41			De novo^a^	Pathogenic	n
33	M	g.53267398C>G	c.3206G>C, p.(Arg1069Pro)	34	Tolerated/probably damaging	PH	Maternal	Likely pathogenic	n
34	M	g.53267325A>C	c.3277+2T>G, p.?	24.2			De novo^a^	Pathogenic	n
35	M	g.53267322C>T	c.3277+5G>A, p.?	19.41			Maternal	Likely pathogenic	n
36	F	g.53265677G>T	c.3278C>A, p.(Ser1093*)	42			De novo^a^	Pathogenic	n
37	M	g.53265568G>T	c.3387C>A, p.(Tyr1129*)	36			De novo	Pathogenic	^27^
38	M	g.53265522G>A	c.3433C>T, p.(Arg1145*)	37			De novo^a^	Pathogenic	n
39	F	g.53265522G>A	c.3433C>T, p.(Arg1145*)	37			Not detected in mother, father unavailable	Likely pathogenic	n
40	F	g.53265009del	c.3457del, p.(Arg1153Glyfs*244)	–			De novo^a^	Pathogenic	n
41	F	g.53263829dup	c.4039dup, p.(Ala1347Glyfs*40)	–			De novo^a^	Pathogenic	n
42	F	g.53263467del	c.4401del, p.(Gly1468Alafs*27)	–			Not detected in mother, father unavailable	Likely pathogenic	n
43	M	g.53272651C>A	c.2752G>T, p.(Val918Phe)	34	Deleterious/possibly damaging	SEC7	Maternal	VUS	n
44	F	g.53265540C>T	c.3415G>A, p.(Ala1139Thr)	32	Tolerated/probably damaging	C-ter	Affected sister also has thepathogenic variant, an affected brother deceased (carrier status unknown); parents unavailable	VUS	n
45	M	g.53265003G>A	c.3463C>T, p.Arg1155Trp	32	Deleterious/probably damaging	C-ter	Maternal	VUS	n
46	M	g.53265003G>A	c.3463C>T, p.Arg1155Trp	32	Deleterious/probably damaging	C-ter	Maternal	VUS	n
47	F	g.53310692del	c.737+10385del, p.?	8.355			Maternal	VUS	n

*ACMG* American College of Medical Genetics and Genomics, *VUS* variant of uncertain significance

^a^Indicates that paternity and maternity have been confirmed

**Table 2:** Summary and comparison of main developmental milestones and epilepsy data in males and females with *IQSEC2* variants

Developmental milestones and epilepsy				
	Males	Females	All	*p* value, test
Number of patients with *IQSEC2* pathogenic variants	22	20	42	
Mean/median age at the study in years	10.2/10.7	13/11	11.6 / 11.0	
**Motor milestones**				
Age at sitting unsupported in months: mean/median age (number)	20.2/17.5 (*n* = 16)	10.2/9.75 (*n* = 12)	15.9 / 12.3 (*n* = 28)	*p* = 0.0015, Wilcoxon–Mann–Whitney test
Age at walking unsupported in months: mean/median age (number)	30.3/28.3 (*n* = 6)	24.5/23 (*n* = 15)		
Unable to walk unsupported	16	1	17	*p* = 4.9E-5, Fisher's exact test
**Language development** (age >3 years)				
Age at first words in years: mean/median age (number)	NA (*n* = 2)	1.2/1.05 (*n* = 8)		
Nonverbal patients	20/22	4/19	24	*p* = 6.2E-6, Fisher's exact test
Speaks several words	0	7	7	
Associates words	2	3	5	
Builds sentences	0	5	5	
**Use of hands** (number)				
Purposeful use of hands	10/21	14/17	24	*p* = 0.043, Fisher's exact test
Absent use of hands	7/21	1/17	8	*p* = 0.053, Fisher's exact test
Hand stereotypies	15/21	8/17	23	*p* = 0.18, Fisher's exact test
**Severity of intellectual disability** (number)				
Mild	0	2	2	
Mild–moderate	0	1	1	
Moderate	0	3	3	
Moderate–severe	0	3	3	
Severe	12	8	20	
Severe–profound	2	0	2	
Profound	8	0	8	
**Behavior** (number)				
Behavioral disturbances	16	13	29	
Autistic behaviors	12	8	20	
Self-injurious behaviors	4	4	8	
**Epilepsy**				
Patients with epilepsy (number)	21	14	35	*p* **=** 0.04, Fisher's exact test
Seizure onset: mean median age (number)	23.9/23.5 (*n* = 20)	66.4/36 (*n* = 13)	40.6 / 34 (*n* = 33)	*p* = 0.1, Wilcoxon–Mann–Whitney test
**Seizure types** (number)				
Documented for: number	19	13	32	
Atypical absences	11	6	17	*p* = 0.72, Fisher's exact test
Generalized tonic–clonic seizures	7	6	13	*p* = 0.72, Fisher's exact test
Tonic seizures	8	4	12	*p* = 0.71, Fisher's exact test
Atonic seizures	11	0	11	*p* = 0.0006, **Fisher's exact test**
Myoclonic seizures	9	1	10	*p* = 0.023, **Fisher's exact test**
Infantile spasms	8	1	9	*p* = 0.049, **Fisher's exact test**
Clonic	2	2	4	
Dyscognitive	1	3	4	
**Resistance to AETs**	13/16	2/11		*p* = 0.002, Fisher's exact test

*AETs* antiepileptic treatments

Manner of hand use was known for 38 patients. Females showed a purposeful use of hands more frequently than males although hand stereotypies were observed in both groups (Table 2). Behavioral issues were reported for 29 patients (13 females and 16 males), including autistic behaviors in 20 (8 females and 12 males), self-injurious behaviors in 8 (4 females and 4 males), temper tantrum, and hyperactivity. The behavior of 7 patients was considered as nonproblematic. Other issues were sleep disorders (early awakening, insomnia) reported in 7 patients and feeding difficulties (poor chewing, regurgitations) mentioned in 10 patients, requiring gastrostomy-tube feeding in 3 patients.

### Epilepsy

Overall, 35/42 patients had epilepsy, including 14/20 (70%) females and 21/22 (95%) males (*p* = 0.04, Fisher’s exact test). Patients without seizures were mainly females (6/7) with mild (*n* = 1) to severe (*n* = 1) ID. In epileptic patients, first seizures were noticed at a mean age of 40.6 months. Females had on average a later age at seizure onset (66.4 months versus 23.9 months in males) although the difference was not statistically significant (Table 2, Fig. 3b).

Polymorphic seizures were observed in most patients. Two male patients had no convulsive seizures but EEG showed continuous or subcontinuous spikes–waves during sleep. The most frequent seizure types were, by descending order of frequency: atypical absences (*n* = 17), generalized tonic–clonic seizures (*n* = 13), tonic seizures (*n* = 12), atonic seizures (*n* = 11), myoclonic seizures (*n* = 10), spasms (*n* = 9), dyscognitive and clonic seizures (*n* = 4). Atonic seizures, spasms, and myoclonic seizures occurred more frequently in males than in females (Table 2). EEG recordings were characterized by a slowing of the background activity (*n* = 16/30), with multifocal (*n* = 8), focal (*n* = 6), or generalized (*n* = 8) spikes and/or spikes–waves, or with bursts of focal slow waves (*n* = 5). Hypsarrhythmia was considered in four patients.

Factors triggering seizures were reported in ten patients, including fever (*n* = 5), sleep (*n* = 4), contact with water and emotions (*n* = 1 each). The epilepsy was classified as infantile spasms in six patients, Lennox–Gastaut syndrome in four, focal epilepsy in four others and unclassified in the others. Status epilepticus was infrequent and mentioned in five patients, including four females. Although seizure frequency was variable, ranging from one per year to dozens per day, most patients had multiple daily seizures (6/12 females and 12/16 males). Responses to antiepileptic treatments (AETs) were variable, regardless of the treatment. The epilepsy was responsive to AETs in 11/28 patients, pharmacoresistant in 15/28, and partially responded in 2/28. Seizures were more frequently pharmacoresistant in males (*n* = 13/16) than in females (*n* = 2/11; *p* = 0.002, Fisher’s exact test; Table 2, Fig. 3c).

There was no correlation between the age at seizure onset and the response to AETs. Likewise, there was no obvious correlation between the age at seizure onset and the severity of ID, because seizures started before 3 years in one of three patients with mild or mild/moderate ID while seven patients with severe to profound ID had a first seizure after the age of 3 years (Fig. 3d). Of note, two patients showed regression of acquisitions, associated with seizure onset in one.

### Clinical examination

Neurological examination data were available for 36 patients including 14/17 (1 female and 13 males) who had not achieved independent walking. Hypotonia, global or truncal, was the most frequently reported sign (*n* = 18; 11/14 nonambulatory and 7/22 ambulatory patients), followed by signs of corticospinal tract involvement (*n* = 8 in both groups, with spasticity of limbs in 4/8), poor or absent eye contact (*n* = 8 noticed in nonambulatory patients only), clumsy/broad-based gait (*n* = 5, all ambulatory), and dystonia (*n* = 2). Nine ambulatory patients had normal neurological examinations.

Two of 34 patients had short stature. Occipitofrontal circumferences (OFC) were available for 34 patients, with four having microcephaly (OFC from −2.5 to −4 SD, including three males), three having an OFC on −2 SD and all others having an OFC ranging from −1.5 to +1.5 SD. Standard deviations of OFC were not significantly different between males and females (*p* = 0.35, Wilcoxon–Mann–Whitney test).

Strabismus was mentioned in eight patients. Minor dysmorphic features were reported in 23 patients, without suggestions for a specific pattern in *IQSEC2*-related encephalopathy.

Brain imaging (magnetic resonance image [MRI] *n* = 36, computed tomography [CT] *n* = 2) was normal (21 patients) or showed nonspecific abnormalities (18 patients), including mild brain atrophy (*n* = 10), thin corpus callosum, and/or abnormal signal of the cerebral white matter (mainly periventricular T2 hyperintensities or myelination delay).

## Discussion

An increasing number of genes on chromosome X are associated with human disorders manifesting in both sexes. *IQSEC2* is the prototype of X-linked genes where variants were first identified in large families comprising almost exclusively affected males. Recent evidence, however, indicates that affected *IQSEC2* female carriers could be nearly as numerous as affected males.

The epilepsy phenotype associated with *IQSEC2* variants has been previously described from the study of 18 patients recruited in epilepsy cohorts.^25^ We report on in this study 37 novel patients, including 7 without epilepsy. The developmental outcome of patients with and without epilepsy was similar. Furthermore, we observed no correlation between the severity of ID, the age at seizure onset, and the response to AETs. Thus, epilepsy appears to result from a parallel pathophysiological process rather than being the underlying cause of the neurodevelopmental disability.

*IQSEC2* is a complicated gene expressing several isoforms and it was tempting to speculate that the nature of pathogenic changes present in males and females could be different. However, our study shows that variant types and distribution are globally similar in males and females, altering the same longer isoform (NM_001111125.2/ NP_001104595.1) corresponding to the synaptic IQSEC2/BRAG1 protein. Yet, pathogenic variants found in females are mainly of truncating type whereas affected males have both truncating and missense variants, and the missense variants are most often inherited from asymptomatic mothers. Strikingly, no males with an entire *IQSEC2* deletion have been reported so far, suggesting that the absence of all isoforms could be lethal or associated with a yet unrecognized disorder.

Genotype/phenotype correlation study in *IQSEC2*-related encephalopathy should take into account the sex of patients and variant type. Three recurrent truncating variants (p.[Tyr269Thrfs*3], p.[Arg1055*], and p.[Arg1145*]) were associated with severe neurodevelopmental disorder in both males and females. However, the overall comparison of male and female phenotypes revealed that females were globally less affected than males. Although most females had severe ID, only female patients had mild to moderate ID. Our study showed that females can sit unsupported at a younger age, have better language abilities, and a later onset of epilepsy with a better response to AETs. Furthermore, 21/22 (95%) male patients had epilepsy versus 14/20 (70%) female patients, and all males with truncating variants had epilepsy or EEG abnormalities versus 13/18 females (72%). Considering the equal number of truncating variants in both genders, these data suggest that truncating *IQSEC2* variants are generally better tolerated in females than in males.

Our results also suggest that missense variants altering functional IQSEC2 domains, and most particularly IQ and SEC7, may be hypomorphic and better tolerated in females (carrier mothers) due to a compensation by the normal allele and can therefore be inherited over several generations through healthy or mildly affected female carriers.^11,18,37^ Some mutations in the SEC7 domain have previously been shown to significantly decrease the ARFGEF activity of IQSEC2. Interestingly, the role of BRAG1/IQSEC2 on AMPA receptors is independent of its ARFGEF activity,^19^ suggesting that that the pathophysiological mechanisms associated with missense and truncating variants in males and females could be slightly different. Splice-site variants also seem to be better tolerated in females than in males, as exemplified by the splice-site variant reported by Madrigal and collaborators.^30^ This is further indicated by the occurrence of males with splice-site variants inherited from unaffected mothers in our series.

In most X-linked disorders predominantly affecting males, sparing of females is usually attributed to skewed XCI favoring the expression of the normal allele.^6^ Study of XCI patterns in females with *IQSEC2* variants led to inconsistent results, most affected females having a random inactivation pattern while a few others show a tendency to XCI skewing. Furthermore, no obvious correlation between the XCI status and the severity of the phenotype seems to emerge. These results should be interpreted by keeping in mind that *IQSEC2* escapes XCI in humans.^7^ Contrary to some XCI escapee genes, whose expression is increased in females, the expression of *IQSEC2* is globally similar in males and females, suggesting that regulatory mechanisms other than XCI are able to compensate its dosage in females.^7^ Furthermore, biallelic expression of many escapees including *IQSEC2* is inconstant depending on the tissue, and also possibly during development.^7^ Altogether, our results suggest that pathogenic variants lead to a loss-of-function of the longest isoform in hemizygous males and haploinsufficiency of the same isoform in heterozygous females.

The phenotype of female *IQSEC2* patients is close to that associated with variants in *SYNGAP1*, also encoding an important component of the excitatory postsynaptic density.^38^ Interestingly, the alterations of neuronal morphology and accelerated maturation of dendritic spines observed in neurons where *IQSEC2* was knocked-down^16^ are reminiscent of the premature development of dendritic spines observed at synapses of *Syngap1*-deficient mice,^39,40^ suggesting that the pathophysiological mechanisms causing *SYNGAP1* and *IQSEC2* disorders are closely related to each other and both lead to an abnormal development of glutamatergic synapses during a critical developmental window. Maturation of glutamatergic synapses is in particular regulated by a switch in the composition of NMDA receptor (N2B subunit being progressively replaced by N2A subunit) and accompanied by a switch from BRAG1/IQSEC2 to BRAG2/IQSEC1 proteins.^17^ However, contrary to *Syngap1*-haploinsufficient mice in which a premature accumulation of AMPA receptors was observed, the number of AMPA receptors was decreased by knockdown of IQSEC2.^19^ This opposite effect is in accordance with differences existing between RasGap (SYNGAP1) and ArfGef (IQSEC2) function.

In conclusion, our study provides evidence that *IQSEC2* encephalopathy is related to the longest synaptic isoform and that females are on average less affected than males, suggesting that a correct dosage of IQSEC2 protein at the synapse is crucial for normal brain development.

## Electronic supplementary material

Supplementary Figure S1

Supplementary Data

Supplementary Table S1

Supplementary Table S2

Supplementary Table S3
