# Correction: IQSEC2-related encephalopathy in males and females: a comparative study including 37 novel patients

**DOI:** 10.1038/s41436-018-0327-7

**Published:** 2018-10-02

**Authors:** Cyril Mignot, Aoife C. McMahon, Claire Bar, Philippe M. Campeau, Claire Davidson, Julien Buratti, Caroline Nava, Marie-Line Jacquemont, Marilyn Tallot, Mathieu Milh, Patrick Edery, Pauline Marzin, Giulia Barcia, Christine Barnerias, Claude Besmond, Thierry Bienvenu, Ange-Line Bruel, Ledia Brunga, Berten Ceulemans, Christine Coubes, Ana G. Cristancho, Fiona Cunningham, Marie-Bertille Dehouck, Elizabeth J. Donner, Bénédicte Duban-Bedu, Christèle Dubourg, Elena Gardella, Julie Gauthier, David Geneviève, Stéphanie Gobin-Limballe, Ethan M. Goldberg, Eveline Hagebeuk, Fadi F. Hamdan, Miroslava Hančárová, Laurence Hubert, Christine Ioos, Shoji Ichikawa, Sandra Janssens, Hubert Journel, Anna Kaminska, Boris Keren, Marije Koopmans, Caroline Lacoste, Petra Laššuthová, Damien Lederer, Daphné Lehalle, Dragan Marjanovic, Julia Métreau, Jacques L. Michaud, Kathryn Miller, Berge A. Minassian, Joannella Morales, Marie-Laure Moutard, Arnold Munnich, Xilma R. Ortiz-Gonzalez, Jean-Marc Pinard, Darina Prchalová, Audrey Putoux, Chloé Quelin, Alyssa R. Rosen, Joelle Roume, Elsa Rossignol, Marleen E. H. Simon, Thomas Smol, Natasha Shur, Ivan Shelihan, Katalin Štěrbová, Emílie Vyhnálková, Catheline Vilain, Julie Soblet, Guillaume Smits, Samuel P. Yang, Jasper J. van der Smagt, Peter M. van Hasselt, Marjan van Kempen, Sarah Weckhuysen, Ingo Helbig, Laurent Villard, Delphine Héron, Bobby Koeleman, Rikke S. Møller, Gaetan Lesca, Katherine L. Helbig, Rima Nabbout, Nienke E. Verbeek, Christel Depienne

**Affiliations:** 1 INSERM, U 1127, CNRS UMR 7225, Sorbonne Universites, UPMC Univ Paris 06 UMR S 1127, Institut du Cerveau et de la Moelle epiniere, ICM, Paris, France; 2 APHP, Hôpital Pitie-Salpetriere, Departement de Genetique et de Cytogenetique; Centre de Reference Deficience Intellectuelle de Causes Rares, GRC UPMC «Deficience Intellectuelle et Autisme», Paris, France; 3 European Molecular Biology Laboratory, European Bioinformatics Institute, Wellcome Genome Campus, Hinxton, Cambridge UK; 4 APHP, Reference Centre for Rare Epilepsies, Necker-Enfants Malades Hospital, Imagine Institute, Paris Descartes University, Paris, France; 5 INSERM U1163, Imagine Institute, Paris, France; 6 Paris Descartes University, Paris, France; 7 Division of Medical Genetics, Department of Pediatrics, CHU Sainte-Justine and University of Montreal, Montreal, QC Canada; 8 CHU La Reunion–Groupe Hospitalier Sud Reunion, La Reunion, France; 9 APHM, Hôpital d’Enfants de La Timone, Service de Neurologie Pediatrique, centre de reference deficiences intellectuelles de cause rare, Marseille, France; 10 Aix Marseille University, INSERM, MMG, UMR-S 1251, Faculte de medecine, Marseille, France; 11 Service de Genetique, Centre de Reference Anomalies du Developpement, Hospices Civils de Lyon, Bron, France; 12 INSERM U1028, CNRS UMR5292, Centre de Recherche en Neurosciences de Lyon, GENDEV Team, Universite Claude Bernard Lyon 1, Bron, France; 13 Claude Bernard Lyon I University, Lyon, France; 14 APHP, Service de genetique medicale, Necker- Enfants Malades Hospital, Imagine Institute, Paris Descartes University, Paris, France; 15 APHP, Unite fonctionnelle de Neurologie, Necker-Enfants Malades Hospital, Imagine Institute, Paris Descartes University, Paris, France; 16 APHP, Laboratoire de Genetique et Biologie Moleculaires, Hôpital Cochin, HUPC, Paris, France; 17 Universite Paris Descartes Paris, Institut de Psychiatrie et de Neurosciences de Paris, Inserm U894, Paris, France; 18 FHU-TRANSLAD, Universite de Bourgogne/CHU Dijon, Dijon, France; 19 INSERM UMR 1231 GAD team, Genetics of Developmental disorders, Universite de Bourgogne-Franche Comte, Dijon, France; 20 Division of Neurology, Department of Paediatrics, The Hospital for Sick Children, University of Toronto, Toronto, ON Canada; 21 Department of Pediatric Neurology, University Hospital and University of Antwerp, Antwerp, Belgium; 22 Departement de Genetique Medicale, Maladies rares et Medecine Personnalisee, CHU de Montpellier, Montpellier, France; 23 Division of Neurology, Children’s Hospital of Philadelphia, Philadelphia, PA USA; 24 Centre de Genetique Chromosomique, Hôpital St-Vincent-de-Paul, GHICL, Lille, France; 25 CHU Rennes, Service de Genetique Moleculaire et Genomique, Rennes, France; 26 Danish Epilepsy Centre Filadelfia, Dianalund, Denmark; 27 Institute for Regional Health Services, University of Southern Denmark, Odense, Denmark; 28 INSERM, U1183 Montpellier, France; 29 Stichting Epilepsie Instellingen Nederland, SEIN, Zwolle, The Netherlands; 30 Department of Biology and Medical Genetics, Charles University 2nd Faculty of Medicine and University Hospital Motol, Prague, Czech Republic; 31 APHP, University Hospital of Paris ïle-de-France ouest, Raymond Poincare Hospital, Garches, France; 32 Department of Clinical Diagnostics, Ambry Genetics, Aliso Viejo, CA USA; 33 Centre for Medical Genetics Ghent, Ghent University Hospital, C. Heymanslaan 10, Ghent, Belgium; 34 Service de Genetique Medicale, Hôpital Chubert, Vannes, France; 35 APHP, Department of Clinical Neurophysiology, Necker-Enfants Malades Hospital, Paris, France; 36 Department of Genetics, University Medical Center Utrecht, Utrecht, The Netherlands; 37 Departement de Genetique Medicale, APHM, Hopital d’Enfants de La Timone, Marseille, France; 38 Child Neurology Department, 2nd Faculty of Medicine, Charles University and Motol Hospital, Prague, Czech Republic; 39 Centre de Genetique Humaine, Institut de Pathologie et de Genetique, Gosselies, Belgium; 40 Unite fonctionnelle de genetique clinique, Centre Hospitalier Intercommunal de Creteil, Creteil, France; 41 APHP, Service de neurologie pediatrique, Hôpital Universitaire Bicetre, Le Kremlin-Bicetre, France; 42 Department of Pediatrics, Albany Medical Center, Albany, NY USA; 43 APHP, Hôpital Trousseau, service de neuropediatrie, Paris, France; 44 Sorbonne Universite, GRC n°19, pathologies Congenitales du Cervelet-LeucoDystrophies, APHP, Hôpital Armand Trousseau, Paris, France; 45 Division of Neuropediatrics, CHU Raymond Poincare (APHP), Garches, France; 46 Service de Genetique Medicale, CLAD Ouest CHU Hôpital Sud, Rennes, France; 47 Unite de Genetique Medicale, Centre de Reference des Maladies rares du Developpement (AnD DI Rares), CHI Poissy–St Germain en Laye, Poissy, France; 48 Departments of Pediatrics and Neurosciences, CHU Sainte-Justine and University of Montreal, Montreal, Canada; 49 Institut de Genetique Medicale, CHRU Lille, Universite de Lille, Lille, France; 50 Department of Genetics, Hôpital Universitaire des Enfants Reine Fabiola, ULB Center of Human Genetics, Universite Libre de Bruxelles, Brussels, Belgium; 51 Department of Genetics, Hôpital Erasme, ULB Center of Human Genetics, Universite Libre de Bruxelles, Brussels, Belgium; 52 Interuniversity Institute of Bioinformatics in Brussels, Universite Libre de Bruxelles, Brussels, Belgium; 53 Clinical Genomics & Predictive Medicine, Providence Medical Group, Dayton, WA USA; 54 Department of Metabolic Diseases, Wilhelmina Children’s Hospital, University Medical Center, Utrecht, The Netherlands; 55 Neurogenetics Group, Center of Molecular Neurology, VIB, Antwerp, Belgium; 56 Neurology Department, University Hospital Antwerp, Antwerp, Belgium; 57 IGBMC, CNRS UMR 7104/INSERM U964/Universite de Strasbourg, Illkirch, France; 58 Institute of Human Genetics, University Hospital Essen, University of Duisburg-Essen, Essen, Germany

Correction to: *Genetics in Medicine* 10.1038/s41436-018-0268-1; published online 12 September 2018

This article was originally published under Nature Research’s License to Publish, but has now been made available under a CC BY 4.0 license. The PDF and HTML versions of the Article have been modified accordingly.

