# Supplementary figures and images for: IQSEC2-related encephalopathy in males and females: a comparative study including 37 novel patients
Source: Genet Med. 2018 Sep 12;21(4):837–49. doi: 10.1038/s41436-018-0268-1 (PMC6752297; doi:10.1038/s41436-018-0268-1)

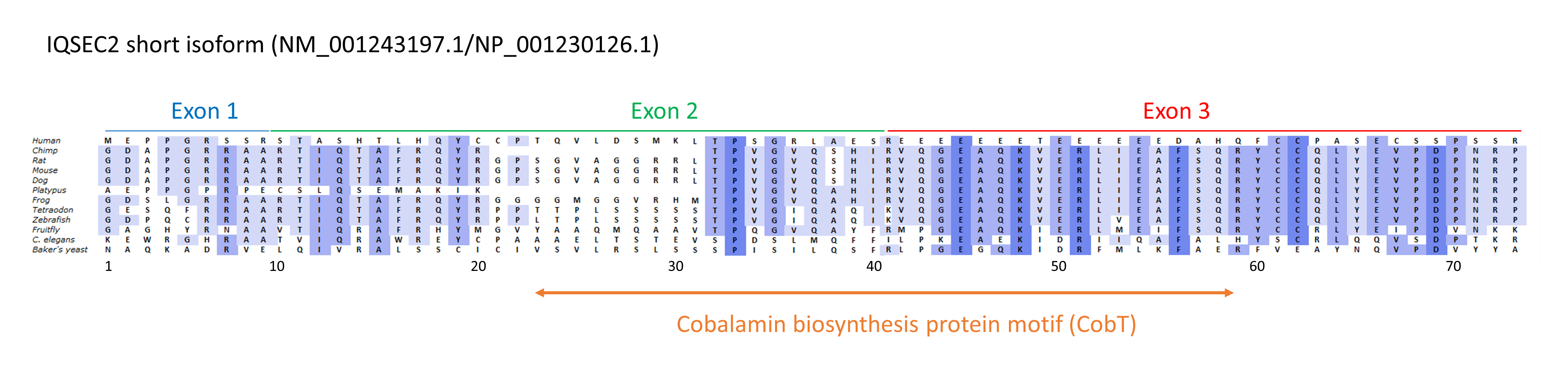

Supplement: Supplementary file 1 — Supplementary Figure S1 [file 41436_2018_268_MOESM1_ESM.tif]
