# Supplementary material for: IQSEC2-related encephalopathy in males and females: a comparative study including 37 novel patients
Source: Genet Med. 2018 Sep 12;21(4):837–49. doi: 10.1038/s41436-018-0268-1 (PMC6752297; doi:10.1038/s41436-018-0268-1)
Supplement: Supplementary file 2 — Supplementary Data [file 41436_2018_268_MOESM2_ESM.docx]

**Supplemental data**

***IQSEC2*-related encephalopathy in males and females: A comparative study including 37 novel patients**

Cyril Mignot^1,2,58*^, Aoife C. McMahon^3^, Claire Bar^4,5^, Philippe M Campeau^6^, Claire Davidson^3^, Julien Buratti^2^, Caroline Nava^1,2,58^, Marie-Line Jacquemont^7^, Marilyn Tallot^7^, Mathieu Milh^8,9,58^, Patrick Edery^10,11,12^, Pauline Marzin^2^, Giulia Barcia^5,13^, Christine Barnerias^14^, Claude Besmond^5^, Thierry Bienvenu^15,16^, Ange-Line Bruel^17,18^, Lieda Brunga^19^, Berten Ceulemans^20^, Christine Coubes^21^, Ana G. Cristancho^22^, Fiona Cunningham^3^, Marie-Bertille Dehouck^23^, Elizabeth J. Donner^19^, Bénédicte Duban-Bedu^23^, Christèle Dubourg^24^, Elena Gardella^25,26^, Julie Gauthier^6^, David Geneviève^21,27^, Stéphanie Gobin-Limballe^13^, Ethan M. Goldberg^22^, Eveline Hagebeuk^28^, Fadi F. Hamdan^6^, Miroslava Hančárová^29^, Laurence Hubert^5^, Christine Ioos^30^, Shoji Ichikawa^31^, Sandra Janssens^32^, Hubert Journel^33^, Anna Kaminska^34^, Boris Keren^2^, Marije Koopmans^35^, Caroline Lacoste^36^, Petra Laššuthová^37^, Damien Lederer^38^, Daphné Lehalle^17,39^, Dragan Marjanovic^25^, Julia Métreau^40^, Jacques L. Michaud^6^, Kathryn Miller^41^, Berge A. Minassian^19^, Joannella Morales^3^, Marie-Laure Moutard^42,43^, Arnold Munnich^5,13^, Xilma R. Ortiz-Gonzalez^22^, Jean-Marc Pinard^44^, Darina Prchalová^29^, Audrey Putoux^10,11,12^, Chloé Quelin^45^, Alyssa R. Rosen^22^, Joelle Roume^46^, Elsa Rossignol^47^, Marleen Simon^35^, Thomas Smol^48^, Natasha Shur^41^, Ivan Shelihan^6^, Katalin Štěrbová^37^, Emílie Vyhnálková^29^, Catheline Vilain^49,50,51^, Julie Soblet^49,50,51^, Guillaume Smits^49,50,51^, Samuel P. Yang^52^, Jasper J. van der Smagt^35^, Peter M. van Hasselt^53^, Marjan van Kempen^35^, Sarah Weckhuysen^54,55,58^, Ingo Helbig^22,58^, Laurent Villard^9,36,58^, Delphine Héron^2^, Bobby Koeleman^35,58^, Rikke S. Møller^24,25,58^, Gaetan Lesca^10,11,12,58^, Katherine Helbig^22,58^, Rima Nabbout^4,5^, Nienke E. Verbeek^35,58^, Christel Depienne^1,56,57,58*^

**Affiliations**

^1^ INSERM, U 1127, CNRS UMR 7225, Sorbonne Universités, UPMC Univ Paris 06 UMR S 1127, Institut du Cerveau et de la Moelle épinière, ICM, F-75013, Paris, France. ^2^ AP-HP, Hôpital Pitié-Salpêtrière, Département de Génétique et de Cytogénétique ; Centre de Référence Déficience Intellectuelle de Causes Rares ; GRC UPMC « Déficience Intellectuelle et Autisme », F-75013, Paris, France. ^3^ European Molecular Biology Laboratory, European Bioinformatics Institute,
Wellcome Genome Campus, Hinxton, Cambridge CB10 1SD, UK. ^4^ APHP, Reference Centre for Rare Epilepsies, Necker-Enfants Malades Hospital, Imagine Institute, Paris Descartes University, Paris, France. ^5^ INSERM U1163, Imagine Institute, Paris, France; Paris Descartes University, France. ^6^ Division of Medical Genetics, Department of Pediatrics, CHU Sainte-Justine and University of Montreal, Montreal, Canada. ^7^ CHU La Réunion - Groupe Hospitalier Sud Réunion, La Réunion, France. ^8^ APHM, Hôpital d'Enfants de La Timone, Service de Neurologie Pédiatrique, centre de référence déficiences intellectuelles de cause rare, Marseille, France. ^9^ Aix Marseille University, INSERM, MMG, UMR-S 1251, Faculté de médecine, Marseille, France. ^10^ Service de Génétique, Centre de Référence Anomalies du Développement, Hospices Civils de Lyon, Bron, France. ^11^ INSERM U1028, CNRS UMR5292, Centre de Recherche en Neurosciences de Lyon, GENDEV Team, Université Claude Bernard Lyon 1, Bron, France. ^12^ Claude Bernard Lyon I University, Lyon, France. ^13^ APHP, Service de génétique médicale, Necker-Enfants Malades Hospital, Imagine Institute, Paris Descartes University, Paris, France. ^14^ APHP, Unité fonctionnelle de Neurologie, Necker-Enfants Malades Hospital, Imagine Institute, Paris Descartes University, Paris, France. ^15^ APHP, Laboratoire de Génétique et Biologie Moléculaires, Hôpital Cochin, HUPC, Paris, France. ^16^ Université Paris Descartes Paris, Institut de Psychiatrie et de Neurosciences de Paris, Inserm U894, Paris, France. ^17^ FHU-TRANSLAD, Université de Bourgogne/CHU Dijon, France. ^18^ INSERM UMR 1231 GAD team, Genetics of Developmental disorders, Université de Bourgogne-Franche Comté, Dijon, France. ^19^ Division of Neurology, Department of Paediatrics, The Hospital for Sick Children, University of Toronto, Toronto, Ontario, Canada. ^20^ Department of Pediatric Neurology, University Hospital and University of Antwerp, Antwerp, Belgium. ^21^ Département de Génétique Médicale, Maladies rares et Médecine Personnalisée, CHU de Montpellier, France. ^22^ Division of Neurology, Children’s Hospital of Philadelphia, Philadelphia, PA, USA. ^23^ Centre de Génétique Chromosomique, Hôpital St-Vincent-de-Paul, GHICL, Lille, France. ^24^ CHU Rennes, Service de Génétique Moléculaire et Génomique, F-35033, Rennes, France. ^25^ Danish Epilepsy Centre Filadelfia, Dianalund, Denmark. ^26^ Institute for Regional Health Services, University of Southern Denmark, Odense, Denmark. ^27^ INSERM U1183, Montpellier, France. ^28^ The Epilepsy Institutes of the Netherlands Foundation (SEIN), 8025 BV Zwolle, the Netherlands. ^29^ Department of Biology and Medical Genetics, Charles University 2nd Faculty of Medicine and University Hospital Motol, Prague, Czech Republic. ^30^ AP-HP, University Hospital of Paris ïle-de-France ouest, Raymond Poincaré Hospital, 92380 Garches, France. ^31^ Department of Clinical Diagnostics, Ambry Genetics, Aliso Viejo, CA, USA ^32^ Centre for Medical Genetics Ghent, Ghent University Hospital, C. Heymanslaan 10, 9000, Ghent, Belgium. ^33^ Service de Génétique Médicale, Hôpital Chubert, 56000 Vannes, France. ^34^ APHP, Department of Clinical Neurophysiology, Necker-Enfants Malades Hospital, Paris, France. ^35^ Department of Genetics, University Medical Center Utrecht, 3508 GA Utrecht, The Netherlands. ^36^ APHM, Hôpital d'Enfants de La Timone, Département de Génétique Médicale, Marseille, France. ^37^ Child Neurology Department, 2nd Faculty of Medicine, Charles University and Motol Hospital, Prague, Czech Republic. ^38^ Centre de Génétique Humaine, Institut de Pathologie et de Génétique, Gosselies, Belgium. ^39^ Unité fonctionnelle de génétique clinique, Centre Hospitalier Intercommunal de Créteil, Créteil, France. ^40^ APHP, Service de neurologie pédiatrique, Hôpital Universitaire Bicêtre, Le Kremlin-Bicêtre, France. ^41^ Department of Pediatrics, Albany Medical Center, Albany, NY, USA ^42^ AP-HP, Hôpital Trousseau, service de neuropédiatrie, 75012 Paris, France. ^43^ Sorbonne Université, GRC n°19, pathologies Congénitales du Cervelet-LeucoDystrophies, AP-HP, Hôpital Armand Trousseau, F-75012 Paris, France. ^44^ Division of Neuropediatrics, CHU Raymond Poincaré (AP-HP), Garches, France. ^45^ Service de Génétique Médicale, CLAD Ouest CHU Hôpital Sud, Rennes, France. ^46^ Unité de Génétique Médicale, Centre de Référence des Maladies rares du Développement (AnD DI Rares), CHI Poissy - St Germain en Laye, 78300 Poissy, France. ^47^ Departments of Pediatrics and Neurosciences, CHU Sainte-Justine and University of Montreal, Montreal, Canada. ^48^ Institut de Génétique Médicale, CHRU Lille, Université de Lille, EA7364 RADEME, Lille, France. ^49^ Department of Genetics, Hôpital Universitaire des Enfants Reine Fabiola, ULB Center of Medical Genetics, Université Libre de Bruxelles, Brussels, Belgium. ^50^ Department of Genetics, Hôpital Erasme. ULB Center of Medical Genetics, Université Libre de Bruxelles, Brussels, Belgium. ^51^ Interuniversity Institute of Bioinformatics in Brussels, Université Libre de Bruxelles. Brussels, Belgium. ^52^ Clinical Genomics & Predictive Medicine, Providence Medical Group, 105 W. 8th Ave., Ste. 454-E Spokane, WA 99204, USA. ^53^ Department of Metabolic Diseases, Wilhelmina Children's Hospital, University Medical Center, Utrecht, The Netherlands. ^54^ Neurogenetics Group, Department of Molecular Genetics, VIB, Antwerp, Belgium. ^55^ Neurology Department, University Hospital Antwerp, Belgium ^56^ IGBMC, CNRS UMR 7104/INSERM U964/Université de Strasbourg, 67400 Illkirch, France.  ^57^ Institute of Human Genetics, University Hospital Essen, University of Duisburg-Essen, 45 239 Essen, Germany. ^58^ EuroEPINOMICS RES consortium

* Correspondence should be addressed to Prof. Christel Depienne (christel.depienne@uni-due.de) or Dr. Cyril Mignot (cyril.mignot@aphp.fr)

**
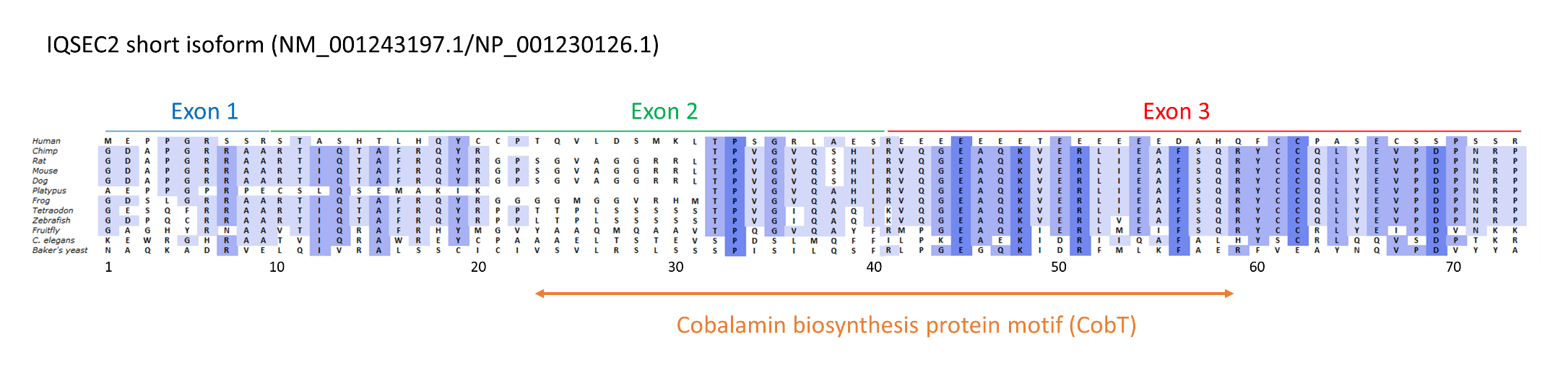
**

**Figure S1 Sequence and characteristics of the 73-amino acid protein encoded by the short *IQSEC2* isoform**. Alignment of the human short NP_001230126.1 protein corresponding to NM_001243197.1 with orthologous sequences, showing that this 73-aa encoded protein has been conserved during evolution (Source: Alamut 2.10) and includes a cobalamin biosynthesis protein motif (CobT, source: MotifFinder; http://www.genome.jp/tools/motif/). This protein does not share any exon in common with the long, synaptic protein (NM_001111125.2/NP_001104595.1, also known in the literature as BRAG1).

**Supplementary table legends**

**Table S1 Individual molecular and clinical data of the 47 patients with *IQSEC2* variants reported in this study.**; IUGR: intrauterine growth retardation, BW: birth weight, BL: birth length; NA: not available or not applicable; y: year(s); m; month(s); w: week(s); d: day(s); sz: seizure(s), GTCS: generalized tonic-clonic seizure, IS: infantile spasms, LGS: Lennox-Gastaut syndrome, NSEE: non-specific epileptic encephalopathy, CSWS: continuous spike-waves during slow sleep; asym.: asymmetrical, SW: spikes-waves; AETs: CBZ: carbamazepine, CLO: clobazam, CLZ: clonazepam, DIA: diazepam, ETH: ethosuximide, FBM: felbamate, HC: hydrocortisone, KD: ketogenic diet, LAC: lacosamide, LEV: levetiracetam, LOR: lorazepam, LTG: lamotrigine, MID: midazolam, NIT: nitrapzeam, OXC: oxcarbamazepine, PHB: phenobarbital, PHE: phenytoin, RUF: rufinamide, STP: stiripentol, TPM: topiramate, VGB: vigabatrin, VNS: vague nerve stimulation, VPA: sodium valproate, ZNS: zonisamide. Medical Exome sequencing refers to sequencing of all coding regions of genes known in human pathology at a given time.

**Table S2 Review of *IQSEC2* variants** **published in the literature and associated phenotypes.** F: females; M: males; ID: intellectual disability; ASD: autism spectrum disorders. All variants are indicated on the long isoform (NM_001111125.2).

**Table S3 Individual sample data used to quantify expression of *IQSEC2* isoforms.** A) Fantom5 Cap Analysis Gene Expression (CAGE) data from the Riken consortium, accessed using the SSTAR portal (TSS expression) http://fantom.gsc.riken.jp/5/sstar/EntrezGene:23096. B) Isoform specific expression was determined by quantifying intron-spanning reads of isoform specific exon-exon boundaries in publically available RNA-seq datasets (accessed through ENA: https://www.ebi.ac.uk/ena).
